# A systematic review of social classroom climate in online and technology-enhanced learning environments in primary and secondary school

**DOI:** 10.1007/s10639-023-11705-9

**Published:** 2023-05-25

**Authors:** Naska Goagoses, Tomi “bgt” Suovuo, Heike Winschiers-Theophilus, Calkin Suero Montero, Nicolas Pope, Erkki Rötkönen, Erkki Sutinen

**Affiliations:** 1 Department of Special Needs Education and Rehabilitation, Carl von Ossietzky University of Oldenburg, Ammerländer Heerstraße 114-118, Oldenburg, 26129 Germany; 2 Department of Computing, University of Turku, Agora, Turku, 20014 Finland; 3 Computer Science Department, Namibia University of Science and Technology, Jackson Kaujeua Street 5, Windhoek, 9000 Namibia; 4 School of Educational Sciences and Psychology, University of Eastern Finland, Yliopistonkatu 7, Futura, Joensuu, 80101 Finland

**Keywords:** Classroom climate, Online learning, Technology, School, Systematic review

## Abstract

Although the significance of a positive social classroom climate in face-to-face learning has been established, its role within online and technology-enhanced learning environments is unclear. The central aim of this systematic review was to synthesize the findings of empirical studies which have examined any aspect of the social classroom climate in online and technology-enhanced learning environments in primary and secondary schools. Appropriate search terms were entered into ACM Digital Library, Web of Science, Scopus, and ERIC in November 2021. Articles were included if they were relevant for the aim, reported primary data, sampled primary/secondary school students and/or teachers, and were published in journals, conference proceedings, or book chapters in English. Furthermore, articles were excluded if they focused on the development/testing of measurement tools. The thematic narrative synthesis includes 29 articles, comprising of qualitative, quantitative, and mixed-method studies. A quality assessment checklist was completed for all. The findings encompass examinations of the social classroom climate in online learning before and during the Covid-19 pandemic, in blended learning environments and a comparison between them. Furthermore, associations between the online social classroom climate and academic variables is explored, as is the fostering thereof through synchronous/asynchronous discussion groups and social media. We discuss the theoretical framing of the studies, the impact of a positive classroom climate in online and technology-enhanced learning environments on students, as well as practical approaches and new opportunities in leveraging technologies. Based on the findings and the studies’ limitations we outline implications and future research, such as the need to consider students’ voices and diversity, technology perspectives, a transdiciplinary approach and the reconceptualization of boundaries.

## Introduction

The significance of a positive classroom climate in primary and secondary schools has been established over the last two decades (Wang et al. , 2020). A positive classroom climate benefits students’ engagement and academic achievement, as well as their social and psychological well-being in face-to-face learning environments (Wang et al. , 2020). However, with a constant reshaping of learning environments through digital technologies, different opinions and findings concerning the disruptiveness, challenges, benefits, and possibilities have been presented. Considering that blended, online and technology-enhanced learning is now the new norm, it is more important than ever that all relevant stakeholders, including educators, researchers, and technology developers, share a common understanding of the classroom climate in this environment, including the benefits and the fostering thereof. To contribute to this development, we set out to conduct a systematic review, narrowing in on studies that have examined the social classroom climate in connection with online and technology-enhanced learning environments in primary and secondary school (i.e., elementary, middle, and high school).

Classroom climate is a multidimensional construct that “represents virtually every aspect of the school experience, including the quality of teaching and learning, school community relationships, school organization, and the institutional and structural features of the school environment.” (p. 315) (Wang and Degol , 2016). Classroom climate can be reduced to four broad categories, namely the academic, which focuses on aspects such as curricula and instruction, institutional, which reflects organizational or structural aspects of the school, safety, which encompasses both physical and emotional security, and community, which focuses on relationships within the school (Wang and Degol , 2016). The community domain is defined by four dimensions, namely quality of interpersonal relationships, connectedness, respect for diversity, as well as community partnerships (Wang and Degol , 2016). Recent conceptualizations of the classroom climate have focused on teacher-student interactions within the classroom, and include the dimensions of instructional support, social-emotional support, as well as classroom organization and management (Wang et al. , 2020). Social-emotional support refers to students’ emotional well-being, including feelings of safety and connectedness, as well as the quality of relationships with teachers and classmates (Wang et al. , 2020). Zurbriggen et al. (2021) state that a good social classroom climate is “characterised by the acceptance of diversity, mutual support, and good social relationships among students” (p. 4.). In this study we use the term “social classroom climate” as encompassing the relational aspects of classroom climate, which include social-emotional and motivational support, as well as student-teacher and peer relationships.

Examining the beneficial effects of a positive social classroom climate, recent reviews have found positive associations with students’ academic achievement, motivation and engagement, self-esteem, social competence, and psychosocial well-being (Thapa et al. , 2013; Aldridge and McChesney , 2018; Wang et al. , 2020; Larson et al. , 2020). Given this plethora of adaptive outcomes, it comes as no surprise that much effort has been dedicated to empirically examining factors that promote a positive classroom climate (Wang et al. , 2020), training pre-service teachers about the importance and possible implementation techniques, and pushing for educational policies and reforms (Velásquez et al. , 2022; Cohen et al. , 2009; Schweig et al. , 2019; ET 2020 Working Group Schools , 2018). Based on studies conducted in face-to-face classes, teachers and educators can foster a positive social classroom climate by being sensitive, responsive, and respectful of social and emotional needs, building relationships that reach beyond school interests, and incorporating students’ perspectives into learning activities (Wang et al. , 2020). Furthermore, school-wide interventions, such as mentoring and school-transition programs, as well as increased opportunities to participate in school improvements, enhanced or correlated with relationships (i.e., as a subdomain of school climate) (Voight and Nation , 2016).

The potential to enhance learning experiences and processes through different technological modes of delivery has previously been recognized, with specific focus on expanding opportunities in space and time (Ahmed and Opoku , 2022). Although a positive classroom climate has been central in face-to-face classes, it is an aspect that has often gone under in research of online and technology-enhanced learning environments. In the past a much greater focus has been placed on academic achievement, motivation, and engagement, whilst the social and emotional aspects of schooling - although continuously being noted as important and missing - have not been adequately investigated (Ahn , 2020). However, current trends in technology and educational research have begun addressing this gap, as have systematic reviews that aim to curate scattered research studies. For instance, a systematic review by Hehir et al. (2021), examining the characteristics of digital resource design to promote student connectedness in higher education, identified the usability, feelings of teacher presence, immediate feedback, synchronicity, and sense of community as central components. Teräs et al. (2020) reflectively identify possible problems that arise from hasty adopting commercial digital learning platforms, as their design is not always driven by established pedagogical practices thereby reducing or even altering concepts of learning and teaching.

Considering the significance of a positive classroom climate and a general trend towards fusing technology with education, the central aim of the current systematic review was to explore and describe empirical studies which have examined the social classroom climate in online and technology-enhanced learning environments in primary and secondary school. In addition to systematically synthesizing the findings of these studies, we set four further objectives with implications for future research and practice. The objectives highlight (1) the relevance of underlying educational and psychological theories in classroom climate research, (2) the impact of a positive social classroom climate in online and technology-enhanced learning environments, in juxtaposition to face-to-face learning environments, (3) practical approaches to fostering a social classroom climate in online and technology-enhanced learning environments, to provide teachers with a preliminary set of practical strategies and techniques, and (4) the identification of new opportunities for leveraging technologies, including important design features and functionalities to promote a positive social classroom climate in online and technology-enhanced learning environments, to adequately inform the design and development of new educational technologies. Implications and future research ideas are outlined, based on the studies methodological limitations, as well as the need to include students’ voices and technology design perspectives in a transdisciplinary approach.

## Methods

### Aims

We set out to conduct a configurative review, which relies on an iterative methodology to generate knowledge and enlightenment by focusing on emerging concepts and valuing uniqueness (Levinsson and Prøitz , 2017; Gough et al. , 2012). The central aim of the current systematic review was to explore and describe empirical studies which have examined the social classroom climate in online and technology-enhanced learning environments, specifically in primary and secondary school. Hence, the the main review question is “What are the major findings of studies that have examined the social classroom climate in connection with online and technology-enhanced learning environments in primary and secondary school?”

To holistically capture the current state of literature, we opted to be open to all research designs and types (i.e., qualitative, quantitative, and mixed-method). Based on the PICo concept (Stern et al. , 2014), we set the following:

**P**opulation: Primary and secondary school students and teachers

Phenomena of **I**nterest: Social classroom climate

**Co**ntext: Online and technology-enhanced learning environments

In addition to describing study findings, we also aimed to gain insights on and discuss the following questions: (1) What theories and models have authors drawn upon to frame their studies?, (2) How does the online social classroom climate impact students?, (3) What practical approaches for fostering a positive social classroom climate in online and technology-enhanced learning environments can be identified?, and (4) What new opportunities are provided through online and technology-enhanced learning environments?

The systematic review was undertaken with the EPPI-Reviewer software, following the PRISMA guidelines (Page et al. , 2021b). A protocol was initially drafted, on which the authors may be contacted for more details.

### Search strategy

To identify studies that focused on the social classroom climate in online learning environments or with technology in primary and secondary school we selected a wide range of search terms. Search terms for online and technology-enhanced learning environments were collected by computer science and educational technology experts in the team (e.g., “remote education”, “distance learning”, “virtual teaching”; n = 104). Search terms for social classroom climate were identified in previous systematic reviews and expanded with relevant terms encompassing specific aspects thereof (e.g., “classroom climate”, “sense of belonging”, “social support”, “teacher-student relations*”, “peer relations*”; n = 31). To exclude studies that did not focus on primary and secondary school we also added search terms to eliminate these (e.g., “higher education”; n = 7). These terms were combined as follows:

(“*remote education*”textsubscript1 *OR* ... *OR* “*virtual teaching*”_104_) *AND* (“*classroom climate*”_1_ *OR* ... ... *OR* “peer relations*”textsubscript31) *NOT* (“*higher education*”_1_ *OR* ... *OR* “*undergraduate*”_7_)

The complete search string can be found in the Supplementary Material (Table S1). The search terms were entered into ACM Digital Library, Web of Science, Scopus, and ERIC in November 2021. The databases were chosen to ensure that we tap at least one major database from the field of information technology, psychology, and education. In the databases, we restricted results by language (English), in line with our eligibility criteria.

### Eligibility criteria

Articles were excluded from the systematic review, if (1) they are not relevant for the topic under investigation, (2) they are not published in English, (3) they are not published in journals, conference paper proceedings, or edited book chapters, (4) they report on secondary or summarized data (e.g. reviews, theoretical papers, descriptive papers without empirical bases), (5) the sample is not primary or secondary school students and/or teachers, and (6) they focused on developing and/or testing measurement tools. Student collaboration, student-teacher interactions, as well as content, instructional, and technological support were not considered to be equivalent with social classroom climate; thus articles focusing on these were excluded.

### Article selection and data extraction

**Fig. 1:**
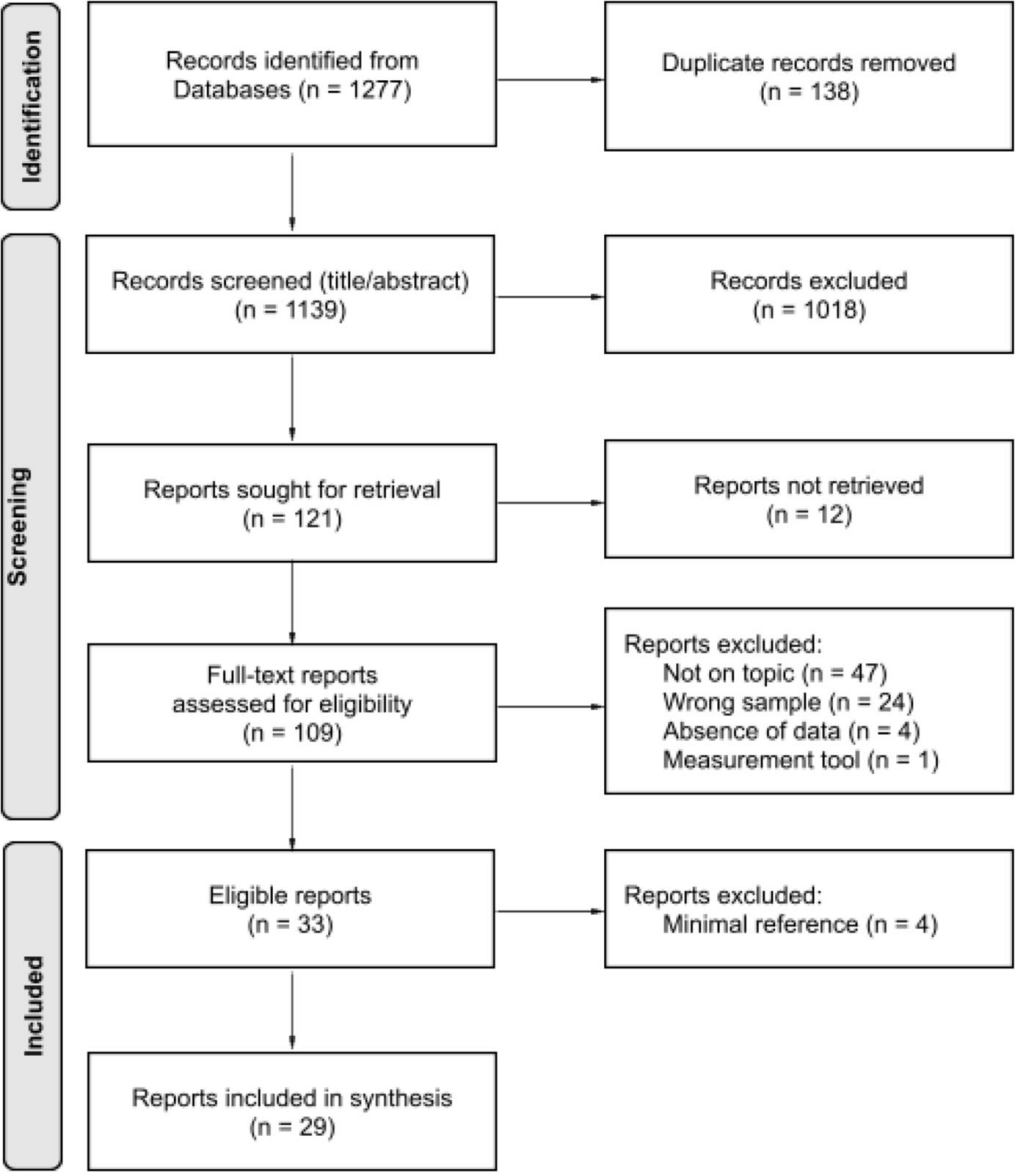
Flow diagram. *Note*. Flow diagram adapted from Page et al. (2021b)

Figure 1 displays the article selection in a flow diagram. After 138 duplicate records were removed, a total of 1139 articles remained. In each step, the eligibility criteria were applied by two independent coders; one with background in educational psychology and one with a background in computer science. For the title and abstract screening, the coders had a 88.8% agreement on the inclusion/exclusion of articles, and for the full-text screening the agreement reached 75.2%. At both stages, the coders discussed the disagreements to make a final consensus-based decision. In the title and abstract screening, simple oversights and misinterpretations (selection errors) were cleared, and a lenient approach to including articles for full-text screening was taken to avoid selection bias. Arisen disagreements at the full-text screening were resolved by the two coders carefully re-reading the entire articles, and conducting an in-depth discussion of the inclusion/exclusion criteria whilst drawing upon their methodological and specialized content expertise. An additional 9 articles were removed after team discussions. Two different coders extracted the data from the remaining 29 articles, and double checked each others work.

### Quality assessment

Assessing the quality of included articles is necessary to establish the presence of methodological issues that could impact the validity and reliability of the findings. The JBI Checklist for Analytical Cross-Sectional Studies was used for quantitative articles, and the Checklist for Qualitative Research for qualitative articles (The Joanna Briggs Institute , 2017). For mixed-method articles we used the Quality Assessment for Diverse studies tool (Harrison et al. , 2021). We opted not to use the results of the quality assessment for the exclusion of articles from the synthesis, but aim to highlight that some findings should be interpreted with caution due to methodological limitations. The quality assessment was completed by two coders, and disagreements were discussed before a summary score was calculated (Hehir et al. , 2021; Ancheta et al. , 2021).

### Synthesis

For the synthesis we drew upon textual narrative and thematic approaches, as these allow for the integration of different types of studies (Dixon-Woods et al. , 2005; Popay et al. , 2006; Barnett-Page and Thomas , 2009; Thomas and Harden , 2008).

In a first step, the results of each article were individually summarized. We narrowed our focus to only present findings that are relevant for our review question. Thus, we only included results that pertained to the social classroom climate or aspects there of (e.g., student-teacher relationships, social-emotional support), i.e., if studies examined/explored additional constructs or phenomena, we did not include these in the summaries. Furthermore, we only reported the results pertaining to primary and secondary school students and teachers; if studies included findings from tertiary education settings, these were not summarized. For quantitative studies, we drew upon findings from regression analyses over correlation analyses. After completing this step, we realized that 4 articles only had minimal reference to the social classroom climate and did not provide informative insights for our review (e.g., studies which simply had one questionnaire item on social relationships, but did not mention this in the theoretical background, the aim of the study, nor in the discussion). Although meeting our inclusion criteria, we collaboratively discussed and opted to exclude these articles from the synthesis.

Next, we explored relationships between the studies, examining similarities in their research aims, focus, contexts, and outcomes. Based on these we inductively categorized the studies into thematically similar groups, which included (1) students’ and teachers’ experiences during the Covid-19 pandemic, (2) teachers’ experiences in established distance education, (3) examinations of blended-learning environments, (4) the usage of social media and chats, (5) comparisons between different environments, and (6) the associations with academic variables. These are reported in the results section, and provide an overview of the major findings of studies that have examined the social classroom climate in connection with online and technology-enhanced learning environment. For the discussion we circle back to the secondary questions we set, abstracting, interpreting, and merging findings from the included studies with additional theoretical and empirical work.

**Table 1:** Characteristics of the included studies

Authors	Country	Sample	Students’ Grade	Research Design (Data Sources)	Hardware	Application(s)
Boling and Beatty (2010)	USA	teacher; 10 students	11	Case study (online postings; informal conversations; observations; interviews)	Laptops	Moodle
Bray et al. (2021)	Ireland	723 teachers; 1004 students	N/P (post-primary school)	Survey (questionnaires)	Unspecified	Unspecified
Casey and Evans (2011)	Australia	1 teacher; 175 students	7-12	Action research (teachers’ planning documents and reflections; field notes; discussions; student work)	Laptops	Ning
Chiu (2021)	China	4 teachers; 426 students	11	Mixed-methods (interviews)	Unspecified	Unspecified
Durgungoz and Durgungoz (2021)	Turkey	1 teacher; 38 students	N/P (students age $=$ 14 years)	Case study (observations; documentation; interviews)	Unspecified	WhatsApp
Ghazinoory and Afshari-Mofrad (2012)	Iran	423 students	N/P (age $=$ 14-19 years, high school students)	Survey (questionnaires)	Unspecified	Unspecified
Harris et al. (2020)	Australia	16 teachers	K12	Case study (focus groups)	Unspecified	Unspecified
Hershkovitz (2018)	Israel	Teachers - Study 2: 3 - Study 3: 111 - Study 4: 66 - Study 7: 4	Study 2: 7-9 Study 3 & 4: N/P (middle/ high school) Study 7: N/P (middle school)	Study 2: case study (observations) Study 3 & 4: survey (questionnaires) Study 7: case study (interviews; observations)	Unspecified	Unspecified
Howley (2021)	Australia, Brazil, China, Ireland, Mexico, New Zealand, South Korea, USA	10 teachers	K12 (including vocational and alternative high school education)	Case study (photovoice; interviews)	Unspecified	Unspecified
Jokić Zorkić et al. (2021)	Serbia	117 teachers; 136 students	N/P (first wave: average age 14.3; second wave: average age 11.0)	Narrative (multi-genre narratives; questionnaires)	Unspecified	Unspecified
Karahan and Roehrig (2016)	USA	22 students	10-12 (alternative education)	Case study (blog posts; observational field notes)	Computers	Ning
Kovacs et al. (2021)	Switzerland	14 teachers	N/P (primary school)	Comparative qualitative inquiry (interviews)	Unspecified	WhatsApp; Teamup; E-mail
Lai (2017)	New Zealand	32 teachers	N/P (secondary school)	Mixed-methods (questionnaires)	Unspecified	Google Hangouts; Unspecified
McKendall et al. (2021)	USA	409 students	9-12	Survey (questionnaires)	Unspecified	West Virginia educational delivery platform
Mælan et al. (2021)	Norway	1755 students (home schooling) 4875 students (regular schooling)	8-10	Survey (questionnaires)	Unspecified	Unspecified
Nowell (2014)	USA	3 teachers; 7 students	N/P (high school)	Narrative (focus groups; interviews; screen images)	Mobile phones; Unspecified	Facebook; Twitter; Edmodo; Instagram; Tumblr; Pinterest
Oren et al. (2002)	Israel	Study 1: 1 teacher, 24 students; Study 2: 11 students	Study 1: N/P (high school) Study 2: 8	Study 1: Case study (online messages) Study 2: Experimental (online messages; questionnaires)	Unspecified	Unspecified
Page et al. (2021a)	Australia	3 teachers	N/P (primary school)	Case study (interviews)	Unspecified	Unspecified
Pirone (2021)	France	5875 teachers (quantitative); 20 teachers (qualitative)	N/P (pre-primary, primary, secondary school)	Mixed-methods (questionnaires; interviews)	Unspecified	Unspecified
Primdahl et al. (2021)	Denmark	8 teachers	N/P (students’ age 12-19 years)	Intervention study (interviews)	Unspecified	Unspecified
Rice and Carter (2015)	USA	4 teachers	N/P (K12)	Narrative (research conversations; interaction records; field notes)	Unspecified	Unspecified
Sarmento et al. (2020)	Brazil	33 students; 8 students, 6 teachers	N/P (secondary education)	Participatory design/ design research (ethnographic observations; focus groups)	Unspecified	Virtual reality
Smith (2013)	New Zealand	30 students	11 (16-18 year-olds)	Experimental (assessments; questionnaires)	Unspecified	Unspecified
Tannert and Gröschner (2021)	Germany	279 students	5-12	Survey (questionnaires)	Unspecified	Unspecified
Vidergor and Ben-Amram (2020)	Israel	27 students	9-10	Case study (learning logs; interviews)	Computers; Mobile phones	Khan Academy platform
Wang et al. (2021)	China	Study 1: 30 teachers Study 2: 1409 students	Study 1: N/P (elementary and secondary schools) Study 2: 4-6	Study 1: Case study (interviews) Study 2: Survey (questionnaires)	Study 1: Unspecified Study 2: Phones; Television; Computers; Tablets	Study 1: Unspecified Study 2: Ding Talk; QQ; WeChat; Tencent Meeting; City Cloud Classroom; Changyanxiaoxue; School Chat; Small Box Homework
Wong (2019)	China	150 students	N/P (secondary school)	Mixed-methods (questionnaires; focus groups)	Unspecified	Google Classroom; E-Mail
Yang et al. (2022)	China	1550 students	7-9	Survey (questionnaires)	Unspecified	Unspecified
Ye et al. (2021)	China	733 students	7-9	Survey (questionnaires)	Unspecified	Unspecified

*Note* Sample: we only report the sample relevant for the current review question; N/P: information not provided in the article; Unspecified: a clear statement regarding the utilized hardware and/or applications was not made in the methods section, nor were these systematically assessed and/or reported in the results sections

**Table 2:** Summary score of studies based on quality assessment checklists

$\varvec{\le .49}$	$\varvec{.50-.69}$	$\varvec{\ge .70}$
Bray et al. (2021)	Durgungoz and Durgungoz (2021)	Boling and Beatty (2010)
Casey and Evans (2011)	Karahan and Roehrig (2016)	Chiu (2021)
Ghazinoory and Afshari-Mofrad (2012)	Mælan et al. (2021)	Harris et al. (2020)
Hershkovitz (2018)	Oren et al. (2002)	Howley (2021)
McKendall et al. (2021)	Page et al. (2021a)	Jokić et al. (2021)
Pirone (2021)	Sarmento et al. (2020)	Kovacs et al. (2021)
Tannert and Gröschner (2021)	Wong (2019)	Lai (2017)
	Yang et al. (2022)	Nowell (2014)
		Primdahl et al. (2021)
		Rice and Carter (2015)
		Smith (2013)
		Vidergor and Ben-Amram (2020)
		Wang et al. (2021)
		Ye et al. (2021)

**Table 3:** Explicitly mentioned theoretical frameworks

Author(s)	Theories/Models
Boling and Beatty (2010)	Cognitive Apprenticeship Model
Casey and Evans (2011)	Social Constructivist Theory; Chaos Theory; Complexity Theory
Chiu (2021)	Self-Determination Theory
Durgungoz and Durgungoz (2021)	Social Presence Theory; Community of Inquiry Framework; Media Richness Theory
Ghazinoory and Afshari-Mofrad (2012)	Technology Acceptance Model
Harris et al. (2020)	IMPACT model
Lai (2017)	Self-Determination Theory; Knowledge Building Community Model
Primdahl et al. (2021)	Caringscape and Carescape Framework
Nowell (2014)	Theory of Media Uses and Gratifications
Tannert and Gröschner (2021)	Control-Value Theory of Achievement Emotions
Vidergor and Ben-Amram (2020)	Zone of Proximal Development Theory
Wang et al. (2021)	Community of Inquiry Framework
Wong (2019)	Self-Determination Theory; Self-Efficacy Theory; Stages of Psychosocial Development (Theory); The Johari Window (model of interpersonal awareness)
Yang et al. (2022)	School-Family-Community Partnership Model (Framework of Six Types of Involvement); Model of Associations between Context, Engagement, and Student Outcomes
Ye et al. (2021)	Process-Person-Context-Time Model (Ecological Systems Theory); Choice Theory

*Note*. We report only explicitly mentioned theoretical models and frameworks; those dealing with designs, instruments, or analysis are not reported, nor those that are implicitly implied by the inclusion of specific constructs

## Results

Table 1 provides a summary of the 29 articles included in the thematic synthesis. The articles were published from 2002 to 2021. Table 2 presents the quality assessment summary ratings for the 8 quantitative, 15 qualitative studies, and 6 mixed-method studies, respectively (Supplementary Tables S2-S5 provide detailed ratings). The articles encompassed samples from the geographic regions Asia (n = 10), Europe (n = 7), North America (n = 5), South America (n = 1), and Oceania (n = 5); as well as one study which includes participants from each previously named region. Overall, the studies reflect a diverse range of students attending primary and secondary school (e.g., special educational needs, minorities, disadvantaged, high achieving, etc.). Table 3 lists the explicit theories and models authors have mentioned within their articles.

### Experiences during the Covid-19 pandemic

School closures related to the Covid-19 pandemic, propelled students and teachers worldwide into online learning environments. This prompted multiple studies focused on assessing subjective experiences related to the classroom climate, including challenges and concerns, as well as adaptations, suggestions, and opportunities.

Analysing the narratives of students and teachers, Zorkić et al. (2021) found disruptions of trust between them. For instance, students reported disruptions in respect, noting teachers expressed anger, lack of patience, or increasing demands. Although students talked about teacher support (e.g., helping with problems, availability), they also mentioned power imbalances. Teachers noted their attempts to maintain caring communication and support, yet also stating that students are lacking in respectful communication and behavior (e.g., politeness, cheating). Based on students and teachers expressions, Zorkić et al. (2021) stated that teachers are encouraged to engage in caring communication and providing social-emotional support that makes students feel safe to repair trust; furthermore, expectations concerning responsibility and conduct should be agreed upon.

Kovacs et al. (2021) found that primary school teachers noted a lack of physical interaction as a concern, that they struggled lost connections, and invested energy to maintain student-teacher relationships. They did this by utilizing “personal videos, story-telling, WhatsApp groups, audio messages, e-mail and to some extent video conferencing” (p. 7558), as well as trying “more “human” encounters” (p. 7558), such as waving to students through windows, exchanging tokens and letters via post, as well as sharing pictures and videos taken at school, in order to bring familiar learning environments to students at home. Howley (2021) examined how the rapid transition to online learning impacted physical education teachers. The teachers reported difficulties in maintaining personal connections with students, as well as establishing relationships with newly enrolled students. The integration of the physical environment was utilized by some teachers to overcome these difficulties (e.g., sharing pictures of local landscapes, meeting in outdoor spaces). Furthermore, the teachers mentioned their concern and support for their students’ social and emotional well-being and development; they specifically noted their continued efforts to have conversations with the whole class or in small groups.

Drawing on questionnaire and interview data, Pirone (2021) found that teachers provided psychological support, focused on listening, reassuring, and motivating students and families. The majority of teachers reported that they contacted students individually at least once per week by email and/or telephone, and that they thought it was important to maintain a strong connection with all students (not just those who were struggling). Interestingly, teachers reported an increased sense of emotional and relational commitment, and an increased sense of closeness with students and families. Furthermore, student initiatives for horizontal learning practices which included providing mutual assistance and maintaining social connections was also elaborated. Similar practices were reported in a study by Wang et al. (2021), in which almost all teachers tried to engage students in online learning by creating a supportive environment. They noted that a relaxed and friendly environment (like a home), that promotes positive emotions, interactions, and cohesion was particularly important whilst students were in quarantine or isolating, as they cannot focus on learning whilst experiencing negative emotions. They further noted the importance of connecting and interacting with classmates, explaining that they often assigned group work to foster this. Teachers also supported the students by providing timely and individualized feedback for homework, and helping students regulate their learning. These practices align well with students’ suggestions for support, who have mentioned the desire for more student-teacher interactions, more feedback and higher availability from teachers, teachers to foster more interactions amongst peers and help with social-emotional aspects of learning (Ye et al. , 2021).

Primdahl et al. (2021) interviewed teachers which were involved in a project that aims to promote mental and social well-being of migrant and refugee students; this includes facilitating social closeness amongst students to encourage emotional and academic support. Several teachers reported difficulties in providing care and social support during the Covid-19 pandemic, and emphasized prioritizing student-teacher relationships, by regularly checking in on the students. They installed several new social media applications (e.g., Facebook, Snapchat) with which the students were familiar, as these offered more friendly, informal communication (e.g., with emojis). However, teachers also noted the difficultly in creating one shared virtual classroom, and that individual (phone) calls were often used. Teachers thought that being together with the students physically was a prerequisite for building relationships, and especially due to language barriers, face-to-face classes allowed for better non-verbal communication; for instance, the assessment of students’ well-being and understanding of assignments via their body language and/or facial expressions. Similarly, feedback was not deemed as having the same quality, with one teacher noting that sending a greeting or praise via messenger was not the same as giving a smile or a pat on the back. Lastly, teachers also recognized the importance of social support between students, perceiving many new arriving students to be socially isolated during the pandemic; however, they were unable to find facilitation measures that were not too difficult and time consuming. The notion of isolation, was also brought forth in a study by Page et al. (2021a), who interviewing teachers, found that one of the challenges reported for students with special educational needs was the disconnection with peers. Namely, that these students may encounter the risk of becoming more socially isolated from peers when they are not able to learn alongside them in physical settings. However, one teacher noted that the students connected outside of class time, via social media or playing games with headsets/microphone.

### Experiences in established distance education

Quite different to the sudden and compulsory move to online learning during the pandemic, studies have also examined the social classroom climate in previously established distance education programs. Again, teachers’ subjective experiences center around challenges and practical solutions.

Harris et al. (2020) found that teachers named the building of relationships as one strategy to support student engagement in compulsory distance education. The teachers described that they built relationships via informal and casual interactions (e.g., chatting one-on-one, calling home). They noted how taking an interest in the students’ lives showed care and helped make the lessons more relevant. Similarly, Lai (2017) examined e-teachers pedagogical practices regarding the development of positive student-teacher relationships. The teachers were concerned with how to develop good relationships and acknowledged that developing a relationship is different online than on-site. They noted that on-site they know the students better, see them more, and are able to check in on their whole lives. Due to the physical distance it is harder to develop a relationship online and it takes more time. One teacher noted that a one-hour video conferencing class is not enough to build a relationship, and many reiterated the importance of keeping regular contact, to remind them the students that they are not alone and the teachers care about them. The majority used communication technologies and social media to develop relationships, and relied on frequent contact and spending time to get to know each other. One teacher stated that they start at the beginning of the school year, by doing fun things, talking about themselves, and commenting on provocative topics. The teachers also noted the importance of developing a class community, which was mainly done through collaborative learning activities.

Rice and Carter (2015) examined how teachers working with special education needs students in virtual schools pursue relationships and the connection with their happiness in teaching. In their narrative accounts, teachers reported the importance of including parents who are physically present with the student to mediate conversations, as they can explain, help with nervousness, and report back on facial expressions and movement; although teachers noted that not all parents were helpful. Teachers recounted the need for frequent communication and contact, noting that this should not only be initiated by teacher and that they aimed to be open and encourage students to also reach out to them (even outside of school hours). Lastly, teachers invited students to physical activities outside of class, and often advocated for them.

### Blended-learning

Studies have also explored the social classroom climate in blended-learning (hybrid) classes, in which students receive technology-mediated materials and opportunities in addition to taking part in face-to-face classroom practices and interactions.

Examining the basic psychological need of relatedness (see Self-Determination Theory Ryan and Deci (2017)), Wong (2019) found that students reported only moderate levels within blended-learning. However, within focus group interviews students mentioned that blended-learning helped them with their need for relatedness. One student stated that they don’t like talking much and now have the opportunity to text chat with others, and another noted that they interact with others they would normally not interact with. Furthermore, students stated that there was less miscommunication, broader ways of understanding student-teacher relationships, and possibilities showcase personal skills, which improved relationships and positive emotions. Also focusing on basic psychological needs, Chiu (2021) altered a learning management system to include aspects of relatedness (amongst other needs) through emotional designs and communications. Students who used this altered system reported more relatedness towards it, than those in the control group (i.e., the same system without the added aspects). Relatedness support from teachers was not found to differ between the groups. Teacher relatedness support was positively associated with all types of engagement, whilst relatedness towards the system was only associated with emotional engagement.

Vidergor and Ben-Amram (2020) found that students referred to the emotional aspects of student-teacher relationships when asked to describe their experiences in the online learning environment (as part of a flipped-classroom approach). They noted that the emotional connection is one of the most important things. They further contrasted what the teacher is able to provide them with what technology never can; this included the teacher caring about them, showing empathy, and providing affectionate feedback (e.g., pep talks). Students also noted that their teacher’s opinion of them and their personal relationship with them, enhances their motivation to learn. Based on participatory design, Sarmento et al. (2020) describe ideas, thoughts, and a prototype that was created by students to enhance the social climate of blended-learning environments. Interviews revealed that the students were dissatisfied with the current climate, the design of the environment, and lack of (digital) innovation. Initial brainstorming had students mention the need for bigger inside and outdoor spaces for socializing, windows to the outside, individual study time and relaxation, better seats, as well as the availability of internet and permission to use mobile phones. The authors summarized the main design concepts for a positive social climate for hybrid learning environments, which included a welcoming atmosphere to release relational tensions between students and teachers, spaces that promote calmness and relaxation, especially in outdoor areas, spaces should be large, open, bright, simplistic, and jovial, enhanced with colors and movable furniture, efficient and usable technology with versatility and customization, and conditions conducive for familiarity, identity, and security.

### Social media and chat functions

The use of social media as an additional learning environment, as well as the use of group chats in blended learning environments, for developing and maintaining student-teacher and peer relationships, have been a significant part in many of the studies, and even the whole focus of some.

Nowell (2014) explored the usage of web 2.0 technologies as relationship building tools within and outside of the classroom. Interviewed teachers stated that they use commercial and education-specific social media to communicate with their students about about homework, school events, but also activities outside of school. This digital support was thought to promote collaborations among students, create a classroom community, and improve relationships within classrooms. The teachers noted that communication via social media works really well, especially for quiet children who usually do not talk much. One teacher mentioned how she asks students to teach her how to use the latest social media applications. Students on the other hand struggled to connect their personal use to education. They stated that they enjoy being passive users of social media, and did not understand why teachers kept separate/private social media accounts. Durgungoz and Durgungoz (2021) examined the usage of a specific social media application, namely WhatsApp, by a teacher and his students. As school and residences were located far away, students mentioned that the online group offered a “meeting point” where they motivated and helped each other with schoolwork. The teacher providing content help made up the majority of correspondence and resulted in feelings of gratitude from the students. The teacher would occasionally reveal information about his current state or location, with the students noting that this made the teacher more like a friend. They also stated how the teachers behavior was different to that in class, i.e., an informal closer relationship in the group than in face-to-face interactions; they enjoyed knowing more and seeing that side of him. Durgungoz and Durgungoz (2021) maintain that these disclosures and communication created a sense of belonging for the students. The teacher also used the group for prompts (check-ins), and encouraged and motivated the students to study. Lastly, the teacher gave students good wishes and prayers (e.g., for holidays and exams), which Durgungoz and Durgungoz (2021) see as important to building an attachment with the teacher, as students feel cared about and noticed.

Investigating the incorporation of social media to enhance motivation and engagement in an alternative school, Karahan and Roehrig (2016), included a session for creating a sense of community in their instructional plan; this included organizing groups, creating profiles, sharing photos/videos, and encouraging connections. Karahan and Roehrig (2016) report that the non-academic features served as fun ice-breakers and increased interactions throughout the year (e.g., posting comments on each others’ pages). Furthermore, the authors note how the online groups are more heterogeneous than in the face-to-face classes (i.e., interactions amongst students from different backgrounds). Reflecting on the use of a social media platform as an additional learning environment, Casey and Evans (2011) recall the surprise of numerous student-directed groups emerging, i.e., groups that did not relate to schoolwork. They note that students enjoyed the connectedness created by the groups, having the opportunity to join, contribute, or lurk within these. Casey and Evans (2011) associate there findings with the importance of peer-relationships in school. Also focusing on group chats, Boling and Beatty (2010) interviewed a teacher and students on asynchronous online discussions in an AP class. The authors note an increased sense of community, with students noting how quickly they started forming new friendships, i.e., with classmates they had known but never really spoken to before. The comments and feedback the students shared let them know each other better and made them feel more comfortable. The authors note how the comments were praiseful and often included a personalized touch, i.e., they were not purely content related. Lastly, the students also stated that they felt the teacher placed trust in them. Also interested in online discussion groups, Oren et al. (2002) examined the development of a social climate in a set of studies. In the first, they explored topics in an asynchronous discussion group which encouraged reading which included a teacher and students from different schools; they found that at the beginning both content and social discussions were held, but with time this dwindled to only content discussions. In the second, they explore the role of moderation and anonymity for synchronous chats; they found that moderation did not influence the amount of content and/or social messages, but more content messages emerged when users were anonymous (i.e., using a nick name) compared to non-anonymous.

### Comparison between environments

Further studies have focused on comparing the social classroom climate between various forms of learning environments, including face-to-face, blended, and purely online formats.

Ghazinoory and Afshari-Mofrad (2012) compared school connectedness between students attending face-to-face schools and those attending smart schools. They found that perceived school connectedness was higher in smart schools than in face-to-face schools. Comparing students perceptions of social connectedness and teacher support, Smith (2013) similarly found that students attending blended-learning classes reported these to be higher than students attending face-to-face classes. Hershkovitz (2018) conducted numerous studies to examine teacher-student relationships in traditional face-to-face versus in technology-enriched face-to-face learning, in which both teachers and students have access to some form of technology. Teachers’ self-reported support (e.g., emotional, instructional) was higher in technology-enriched classes than in traditional ones in two studies, and did not differ in a third. In their last study, they compared teachers descriptions of student-teacher relationships in distance versus face-to-face learning. They found that teachers spoke about academically-motivated students with whom they had intellectual connections in distance classes, and about poorly academically-motivated students with whom a connection arose due to distress in face-to-face classes. Furthermore, teachers mentioned unusual communication in distance classes (e.g., more informative, expressive). Lastly, teachers reported that they care about students academically in distance classes, and care more about students emotionally in face-to-face classes, i.e., distance learning focuses on academic issues and not emotional bonds. Lastly, comparing in-person learning, virtual learning, and blended/hybrid learning in an educational program for disadvantaged (exceptionally driven) students, McKendall et al. (2021) found significant differences perceived availability of help, feedback, and support from teachers. However, the small effect sizes led the authors to conclude that there is only negligible practical significance.

### Associations with academic variables

Mainly conducted during the Covid-19 pandemic, studies have also focused on the associations between an online social classroom climate and relevant academic variables.

These found that perceptions of the general atmosphere in digital lessons and the student-teacher relationship during distance learning was positively correlated with students’ achievement (Tannert and Gröschner , 2021). Furthermore, high-achieving students reported that they received less feedback from teachers in homeschooling (i.e., remote learning during the Covid-19 pandemic) than low-achieving students; however, the same trend was found in regular schooling (Mælan et al. , 2021). A comparison of the association between students achievement-level and aspects of student-teacher relationships between homeschooling and regular schooling did not reveal uniform results (Mælan et al. , 2021). Furthermore, the general atmosphere and student-teacher relationships showed no association with students’ self-efficacy (Tannert and Gröschner , 2021). Student-teacher relationships did however have a positive direct effect onto students academic engagement during distance learning (Ye et al. , 2021). Nonetheless, having a better student-teacher relationship intensified the association between difficulties with online learning and academic engagement (Ye et al. , 2021). Yang et al. (2022) found that perceptions of teacher support during emergency remote teaching was positively associated with students’ affective engagement. Students’ perceptions of student-teacher relationships were associated with the relevance and enjoyment they ascribed education during remote learning (Bray et al. , 2021), and with feelings of enjoyment and anxiety, whilst the perceived general online atmosphere was not (Tannert and Gröschner , 2021). Clear instructions and communication from the teacher was associated with students feelings of comfort interacting with others during online learning, which was also related to students’ satisfaction with online experiences and cognitive presence (Wang et al. , 2021).

## Discussion

Aligning with our aims, the following section focuses on reflecting and discussing the articles dealing with the social classroom climate in online or technology-enhanced environments. Specifically, we address the theories and models included in the articles, how the online classroom climate impacts students, as well as what practical approaches and new opportunities were identified.

### Theories and models

An additional objective of the current review was to examine which theories and models authors mentioned within their articles, for studies that connect the social classroom climate with online and technology-enhanced learning environments (see Table 3). The Community of Inquiry Framework (Garrison et al. , 2010) and the Self-Determination Theory (Ryan and Deci , 2017) were the most cited, with the latter having been identified as potential theoretical framework for examining the classroom climate by Wang et al. (2020). Previous systematic reviews focused on the classroom climate have also named the Bio-Ecological Theory, Risk and Resilience Model, Attachment Theory, Social Control Theory, Social Cognitive Theory, Stage-Environment Fit Theory, and Systems View of School Climate as unique potential theoretical frameworks (Wang and Degol , 2016; Rudasill et al. , 2018). Given this plethora, it is somewhat surprising that the majority of our included studies have not explicitly mentioned these or any other theories. In their review, Aldridge and McChesney (2018) also noted the general absence of theoretical frameworks and called upon researchers to make these more explicit in future (see also Thapa et al. (2013)). A clear conceptual definition and theoretical framework is also important for the operationalization of classroom climate in research. As there have been multiple recent reviews on the assessment of classroom climate (Rocha et al. , 2019; Lenz et al. , 2021; Marraccini et al. , 2020; Grazia and Molinari , 2021), we will not delve into this in detail; however, it should be noted that these are all based on climate within face-to-face learning environments, thus highlighting the need for more standardized, psychometrically-evaluated assessment tools for online learning environments (e.g., (Kaufmann et al. , 2016; Li, Kong and Chen , 2015)).

### Impact on students

Studies in face-to-face classes have found that a positive social classroom climate has a beneficial impact on a range of academic and social-emotional outcomes for students (Thapa et al. , 2013; Aldridge and McChesney , 2018; Wang et al. , 2020; Larson et al. , 2020; Goetz et al. , 2021). Although the number of studies that have thus far been conducted do not allow for conclusive statements, they do indicate that online social climate has similarly beneficial effects on achievement, engagement, and positive emotions (Tannert and Gröschner , 2021; Ye et al. , 2021; Yang et al. , 2022; Bray et al. , 2021). These studies thus highlight that the social climate remains an important educational factor even within online learning environments. Furthermore, the studies highlight confounding and moderator variables, which include students’ prior achievement level, grade level, gender, socio-economic status (and related factors), twenty-first century skills, and parental involvement. Considering that this is one of the aspects in which many quantitative studies lost quality points (see Section 4.5.2), we urge researchers to pay more attention to these in future.

Another interesting finding is that in the studies which compared traditional face-to-face with technology-enhanced learning environments (e.g., smart schools, blended-learning classes), found that the later had a positive impact on students perceptions of the social classroom climate (Ghazinoory and Afshari-Mofrad , 2012; Smith , 2013; Hershkovitz , 2018). Hershkovitz (2018) propose that teachers reference to an increased use of learner-centered activities, active/independent yet collaborative learning, increased interactions, and increased enjoyment is relevant to student-teacher relationships in technology-enriched learning environments. However, teachers spoke about how online teaching is focused on academic issues and not strengthening emotional bonds. Smith (2013) similarly state that their findings, based on students’ questionnaire responses, was contradictory to the teachers opinion, who noted in a reflective blog that the blended-learning students were becoming less engaged and that “genuine” human connection might be driving engagement in the face-to-face class. The authors note that the teachers (negative) experience with technology-enhanced classrooms should not simply be equated with students’ experiences and perceptions. This aligns with research in face-to-face classes, which has also found differences in perceptions of the classroom climate between teachers and students (Raviv et al. , 1990). Furthermore, studies included in the current review also demonstrate a slant towards qualitatively assessing teachers’ experiences more than that of students. However, as students are equal partners in the creation of positive social classroom climates, it is important that they are involved and consulted for solutions and new ideas.

### Practical approaches

Having to work through limitations within online and technology-enhanced environments, teachers have also reported practical approaches they utilized in order to foster a positive social classroom climate; although further empirical studies are required to examine the effectiveness of these practices, they may offer practical insights to educators. Firstly, teachers mentioned instigating communication and interactions outside the technological realm, such as meeting outdoors or exchanging physical tokens (Kovacs et al. , 2021; Howley , 2021; Rice and Carter , 2015); the connection to a physical space was also created by sharing environmental photos and videos with the students (Kovacs et al. , 2021; Howley , 2021). Previous studies have indicated that the physical classroom environment is important for students’ learning (Weinstein , 1979; Lewinski , 2015), yet a direct connection between physical space and social aspects (beyond arrangements conducive for collaborative work) has not been proposed. Future explorations could consider whether an observed need for physical space is indeed a relevant factor (crystallized through the absence thereof), or merely a manifestation of the need for social presence, or simply a conversation starter.

Teachers also reported the additional need to regularly communicate with students individually (Pirone , 2021; Primdahl et al. , 2021; Harris et al. , 2020; Rice and Carter , 2015). This aligns with previous studies which demonstrate the effectiveness of spending one-on-one time with students, checking in on students, and conducting home visits, for fostering teacher-student relationships (Kincade et al. , 2020). Another strategy reported by teachers was the use of multiple communication technologies and applications to communicate with their students (Primdahl et al. , 2021; Kovacs et al. , 2021); supplementing video conferences with simple phone calls and social media communication was the most common (Primdahl et al. , 2021; Harris et al. , 2020; Lai , 2017; Kovacs et al. , 2021). Lastly, teachers noted the importance of informal conversations, getting to know the students (e.g., their interests), as well as providing psychological, emotional and motivational support (Harris et al. , 2020; Pirone , 2021; Wang et al. , 2021; Lai , 2017). Again this aligns with practices identified to foster teacher-student relationships in face-to-face learning enviornments (e.g., getting to know students, expressing care) (Kincade et al. , 2020). Surprisingly, the aspect of praise, being one of the most effective practices (Kincade et al. , 2020), was only addressed by Boling and Beatty (2010); it is unclear whether praise may have been included in individualized feedback (Wang et al. , 2021; Vidergor and Ben-Amram , 2020).

Although fostering the relationships and supportive interactions between students was addressed (Pirone , 2021; Wang et al. , 2021; Ye et al. , 2021; Primdahl et al. , 2021; Page et al. , 2021a), most did not reveal how this was concretely achieved in online or technology-enhanced learning environments. Teachers merely mentioned the use of group work (Wang et al. , 2021), the integration of social media (Nowell , 2014), and that students find time outside of class to connect (Page et al. , 2021a). Youth generally spend more time online, and view the environment as conducive for maintaining and developing friendships (Mittmann et al. , 2022; Wendt and Langmeyer , 2021). Future studies should explore the adoption of strategies suggested for enhancing online peer networks and support in tertiary education, such as implementing collaborative learning and creating formal peer mentoring programs (Wissing et al. , 2022).

### Leveraging technologies

With the introduction of digital technologies, different features are provided which offer new opportunities to enrich social relations in and beyond classroom activities.

#### Inclusive mingling

An interesting observation, reported by Wong (2019); Boling and Beatty (2010); Karahan and Roehrig (2016), is that online and technology-enhanced learning environments fostered relationships between students that would not normally interact in face-to-face classes. Reported quotations indicate that that this refers to students of different backgrounds (Karahan and Roehrig , 2016), and that in the online platform common topics and interests emerged (Wong , 2019). Students in the study reported by Durgungoz and Durgungoz (2021) indicated that a simple WhatsApp group provided them with a “meeting point” after school, where they could communicate (about academic and non-academic topics) with students that lived further away (i.e., students they would not normally meet after school). Leveraging technologies specifically for this purpose, may provide new opportunities for promoting social inclusion, for instance as a tool for cooperative learning or promoting exchanges outside of the classroom (Juvonen et al. , 2019).

#### Expressive choices

Another new opportunity provided by online and technology-enhanced learning environments is the increased choice students have for expressing themselves and communicating with others; i.e., in face-to-face classes this is usually limited to verbal communication, yet in the digital space this can more readily include written or pictorial communication. Results of the included studies indicate that this can be especially beneficial for students who usually do not talk as much (Wong , 2019; Nowell , 2014). Opening up multiple modes of communication (e.g., talking, writing, drawing, sending emojis) has also been previously suggested as a facilitation technique when working with “verbally shy” primary school students online (Winschiers-Theophilus et al. , 2022). Furthermore, Hershkovitz (2018) reported that students responded quicker and with more details, communicative expressions (e.g., exclamation marks) when writing texts to their teachers.

#### Informal exchange

As noted above in the Section 4.3, teachers mentioned the use of informal exchanges with students (Durgungoz and Durgungoz , 2021; Primdahl et al. , 2021; Nowell , 2014). Students in the study by Durgungoz and Durgungoz (2021) noted that the informal exchanges over social media where different to those in the face-to-face classes, with the former fostering more closer relationships. Hence, although informal exchanges can also take place in face-to-face classes, we believe that this can take on a different form in online learning environments. For one this could be due to additional technical features; for instance the use of emojis (Primdahl et al. , 2021), which has recently been described as a novel form of visual communication with potential in (higher) educational settings (Doiron , 2018). Even amongst the students, the use of social media as an educational tool, was often used for informal exchanges (Karahan and Roehrig , 2016; Casey and Evans , 2011).

### Limitations

#### Limitations of the review process

Although aiming to create an extensive list of search terms, we do acknowledge that articles which may have made reference to specific hardware and applications without embedding the study within the context of online and technology-enhanced learning environments could have been missed. Furthermore, the often blurry and overlapping terms, make it hard to navigate and merge findings. Although defining social classroom climate for the review, a clear conceptual distinction with similar phenomena, variables, tasks, and behaviors, was not always easy to achieve; resulting in multiple discussions during the screening phase. Furthermore, we only included English-language articles, thereby excluding relevant articles that may have been published in other languages. Lastly, we synthesized the findings of studies spanning across 20 years, which includes a wide range of teaching philosophies and best-practices as well as major developments in technology.

#### Limitations of the included studies

The quality assessment revealed that main issues in quantitative studies included an inadequate description of the instruments (including their psychometric properties), confounding factors not being considered, and inadequate statistical analyses. Main issues in qualitative studies included the role/influence of the researcher not being considered, and no reference being made to ethical considerations. Mixed-method studies often did not provide justifications for tools or analyses, and did not involve stakeholders. A further identified limitation is that only half of of the included studies made some reference to a concrete theory or model. As noted previously “Section 4.1”, this is problematic from a research perspective, with educational technology research often being criticised for lacking theoretical foundations (Hew et al. , 2019). Technologies, as well as utilized teaching and facilitation techniques in online and technology-enhanced learning environments should be anchored in relevant educational (and psychological) theories and empirical research (Goagoses et al. , 2022). Lastly, we found that more than half of the included studies, authors did not systematically report and/or assess utilized technologies (hardware and software). Considering that different technologies promote different learning and communication approaches, the actual possibility to promote a social classroom climate varies across platforms and is therefore an important factor to consider.

### Implications and future research

#### Students’ voices and diversity

Qualitative studies included in the current review more often included the perspectives and actions of teachers (n = 13) than those of students (n = 7). Although the promotion of a positive classroom climate is often viewed as the responsibility of the classroom teacher, examining and including students’ perspectives and ideas is vital. This is illustrated in the work by Zorkić et al. (2021), where teacher and student views offer interesting parallels, and Smith (2013) who found contradictions between students’ and teachers’ responses. Students’ perspectives on student-teacher relationships in face-to-face learning environments has provided useful insights for educators (García-Moya et al. , 2020), and should especially be considered in online and technology-enhanced learning environments due to their advanced experience with technology. Hence, we do not simply mean that students’ perceptions of their current climate be considered, but rather - aligning with notions of student-centered learning and agentic engagement - the involvement of students in actively creating and developing pedagogical strategies and educational technology to foster a positive social classroom climate.

It is noteworthy that the studies included in the current review were conducted in diverse geographic regions (see Table 1); however, samples situated in the South American and African continent are still underrepresented. Higher education studies indicate that students’ cultural and linguistic backgrounds impact how they encounter online learning environments (Hannon and D’Netto , 2007), and that although instructional strategies to address cultural diversity can be implemented, may challenges remain (Kumi-Yeboah et al. , 2020). Considering local education systems and globalization, more empirical research is needed with participants with diverse backgrounds to inform future developments in strategies and technologies to enhance the classroom climate in online and technology-enhanced learning environments.

A positive classroom climate is beneficial for the inclusion of diverse students in face-to-face learning environments (Zurbriggen et al. , 2021), and should not be forgotten in online learning. In studies included in the current review, teachers reported on the challenges of creating a positive social classroom climate with special educational needs students or those with language-barriers (Primdahl et al. , 2021; Page et al. , 2021a). Primdahl et al. (2021) and Rice and Carter (2015) report on the importance of facial expressions and body language when working with these students; aspects that often fall short in online learning environments. It is important for future studies to explore fitting technological functions and pedagogical strategies, as well as including students with special educational needs in technology design (Benton and Johnson , 2015).

#### Technology perspectives

Most of the studies concerned teachers trying to find out how and what they can manage with the technologies available. The focus was predominately on the use of provided features, like the management of assignments (submission and feedback) in learning platforms, such as Moodle and Ning (Casey and Evans , 2011), and additional communications in social media (Durgungoz and Durgungoz , 2021; Primdahl et al. , 2021; Nowell , 2014). Sarmento et al. (2020) and Vidergor and Ben-Amram (2020) were the only articles, where the researchers were invested in the design of the used technology, positioning their work in the domain of design science research (Drechsler and Hevner , 2016). Sarmento et al. (2020) reported that students were dissatisfied with the current design of the environment, and lack of (digital) innovation. Furthermore, none of the included studies investigated emerging technologies, such as augmented, mixed, or virtual reality. Major advances have been made in emerging educational technology research and development over the last years and have demonstrated a high potential to enhance online learning experiences. Oren et al. (2002) and Chiu (2021) dissected the design of their applied technology thoroughly and analysed how exactly the design affected the effects on social climate. Hehir et al. (2021), based on a systematic review, established that designing digital tools to support connectedness need to focus on “usability; teacher interaction; immediacy; synchronicity; and community”. Thus, while empirical studies need to inform requirements for future technology developments, at the same time we need to leverage the potential of technologies to enhance learning experiences. McVeigh-Schultz et al. (2018) reminds us of the urgency to no longer attempt to transfer traditional face-to face communications but to rather explore new ways and meanings of social interactions afforded by digital tools, such as for example visualised and animated feedback. Chiu (2021) recommend that in order to promote a positive atmosphere the technology-enhanced learning environment needs to include personal and emotional design and communications. Communication platforms such as Ohyay, offer simple mechanisms to personalise learning environments, as has been demonstrated by an online learning platform co-designed with children (Zaman et al. , 2022). In this light, we maintain that future educational technology development should be further informed by empirical studies, such as the ones reviewed, to promote positive social classroom climate.

#### Transdisciplinarity

While we observe a paucity of exploring emerging technologies in studies concerned with social classroom climate, Pellas et al. (2021), based on a systematic review of virtual reality in education, note a void of studies using educational theories to inform the design of virtual reality applications. This indicates disjoint studies of pedagogical constructs in technology-enhanced contexts on the one side and (emerging) technologies on the other. We maintain that it is equally important for educational researchers to explore emerging technologies as its for technology developers to be informed by educational theories and empirical studies. Moreover, we are concerned with the gap between the vast research and advances in educational technologies and techniques, ranging from technologies supporting special needs to promoting engagement among others, and the lack of use or even awareness thereof in educational practice. Considering the most recent articles, often concerned with challenges of adapting to an online only environment during the pandemic, using ad-hoc approaches, demonstrate little cognisance by educational practitioners and stakeholders of prior research on established distance learning nor on emerging educational technologies. We therefore maintain that a transdisciplinary approach is required to synthesise empirical findings, educational theories and emerging technology developments in order to promote a positive social classroom climate in online and technology-enhanced learning environments, including the different perspectives and experiences of practitioners, educational and technology researchers and developers, besides other stakeholders in the educational context.

#### Reconceptualizing boundaries

Although not always specifically addressed, the teachers’ reports indicate that teachers devoted time outside of the normal classroom hours to offer support and maintain relationships with their students. This aligns with similar trends, reported especially during the Covid-19 pandemic (Pluut and Wonders , 2020), with remote working and technological advances leading to a blurred work-life balance. Within face-to-face learning environments teachers were tasked to address both academic and social-emotional aspects at school, yet within online learning environments there seems to be a displacement of social interactions to other communication tools and out-of-class times. Aspects of the social classroom climate, like student-teacher relationships, offer interesting juxtapositions, as resources and boundaries should be safeguarded, yet the construct itself (and the promotion thereof) often reaches outside of schooling (e.g., home visits (Kincade et al. , 2020), extracurricular activities (Juvonen et al. , 2019)). This similarly applies at the content level, with students and teachers noting the importance of informal exchanges (see Section 4.4.3). Although students are encouraged to share and open-up, teachers have diverging positions on keeping their private and professional lives separate; which may not always be understood by students (Nowell , 2014). Lastly, the move to online and technology-enhanced learning environments also brings about a shift in expertise. Teachers moved to applications with which students are more familiar, and even mentioned that they required students to teach them (Primdahl et al. , 2021; Nowell , 2014). This shift is likely to bring about a reconceptualization of practices that promote a positive student-teacher relationships, such as negotiating respect, proving students with choices and empowerment, as well as sense of responsibility (Kincade et al. , 2020).

## Conclusion

Previous systematic reviews have highlighted the significance of a positive classroom climate in face-to-face environments (Thapa et al. , 2013; Aldridge and McChesney , 2018; Wang et al. , 2020; Larson et al. , 2020) and the current systematic review expands this to online and technology-enhanced learning environments. Synthesizing 29 heterogeneous studies, we present teachers’ and students’ perspectives on challenges and practical approaches in emergency and established distance education, added opportunities of blended and technology-enhanced learning, comparisons between environments, and the impact of a positive social classroom climate on student outcomes. The systematic review provides a configurative overview of the empirical studies on the topic, which may serve as a starting point for educators, researchers, and technology developers in their endeavours to promote a positive social classroom climate within established and continuously evolving online and technology enhanced learning environments. A review of the studies suggests the need for more research converging disciplines, framed in educational theories, considering students’ voices and diversity, leveraging emerging technologies, and re-conceptualizing boundaries.

## Data Availability

Data sharing not applicable to this article as no datasets were generated or analysed during the current review. The data extraction results are not publicly available due to copyright policies.
